# Inter-Laboratory Concordance of Cerebrospinal Fluid and Serum Kappa Free Light Chain Measurements

**DOI:** 10.3390/biom12050677

**Published:** 2022-05-07

**Authors:** Patrizia Natali, Roberta Bedin, Gaetano Bernardi, Elena Corsini, Eleonora Cocco, Lucia Schirru, Ilaria Crespi, Marta Lamonaca, Arianna Sala, Cinzia Nicolò, Massimiliano Di Filippo, Alfredo Villa, Viviana Nociti, Teresa De Michele, Paola Cavalla, Paola Caropreso, Francesca Vitetta, Maria Rosaria Cucinelli, Matteo Gastaldi, Tommaso Trenti, Patrizia Sola, Diana Ferraro, on behalf of RIREMS (Rising Researchers in MS)

**Affiliations:** 1 Department of Laboratory Medicine, Azienda Ospedaliero-Universitaria and Azienda Unità Sanitaria Locale, 41126 Modena, Italy; p.natali@ausl.mo.it (P.N.); m.cucinelli@ausl.mo.it (M.R.C.); t.trenti@ausl.mo.it (T.T.); 2 Department of Biomedical, Metabolic and Neurosciences, University of Modena and Reggio Emilia, 41126 Modena, Italy; roberta.bedin@unimore.it; 3 Laboratory Medicine Unit, Fondazione IRCCS Istituto Neurologico Carlo Besta, 20133 Milan, Italy; gaetano.bernardi@istituto-besta.it (G.B.); elena.corsini@istituto-besta.it (E.C.); 4 Multiple Sclerosis Center, ATS Sardegna/University of Cagliari, 09047 Cagliari, Italy; ecocco@unica.it (E.C.); lucia.schirru@atssardegna.it (L.S.); 5 Clinical Biochemistry Laboratory, Azienda Ospedaliero Universitaria Maggiore della Carità of Novara, 28100 Novara, Italy; ilaria.crespi@maggioreosp.novara.it (I.C.); marta.lamonaca@libero.it (M.L.); 6 Neurology Unit, CReSM, Azienda Ospedaliero Universitaria San Luigi Gonzaga, 10043 Orbassano, Italy; sala.arianna72@gmail.com; 7 Clinical Chemistry and Microbiology Laboratory, Azienda Ospedaliero Universitaria San Luigi Gonzaga, 10043 Orbassano, Italy; cinzia.nicolo@tiscali.it; 8 Neurology Unit, Department of Medicine, University of Perugia, 06132 Perugia, Italy; massimiliano.difilippo@unipg.it; 9 Clinical Pathology and Haematology Laboratory, Azienda Ospedaliera of Perugia, 06132 Perugia, Italy; alfredo.villa@ospedale.perugia.it; 10 Multiple Sclerosis Center, Fondazione Policlinico Universitario “A. Gemelli” IRCCS, Catholic University, 00168 Rome, Italy; viviana.nociti@policlinicogemelli.it; 11 Clinical Chemistry, Biochemistry and Molecular Biology Laboratory, Fondazione Policlinico Universitario “A. Gemelli” IRCCS, 00168 Rome, Italy; teresa.demichele@policlinicogemelli.it; 12 Multiple Sclerosis Center, Department of Neurosciences and Mental Health, Azienda Ospedaliero-Universitaria Città della Salute e della Scienza of Torino, 10126 Torino, Italy; paola.cavalla@unito.it; 13 Clinical Biochemistry Laboratory, Department of Laboratory Medicine, Azienda Ospedaliero-Universitaria Città della Salute e della Scienza of Torino, 10126 Torino, Italy; pcaropreso@cittadellasalute.to.it; 14 Neurology Unit, Azienda Ospedaliero-Universitaria of Modena, 41126 Modena, Italy; vitetta.francesca@aou.mo.it (F.V.); sola.patrizia@aou.mo.it (P.S.); 15 Neuroimmunology Laboratory, IRCCS Mondino Foundation, 27100 Pavia, Italy; matteo.gastaldi@mondino.it

**Keywords:** multiple sclerosis, kappa index, cerebrospinal fluid, free light chains

## Abstract

The kappa index (K-Index), calculated by dividing the cerebrospinal fluid (CSF)/serum kappa free light chain (KFLC) ratio by the CSF/serum albumin ratio, is gaining increasing interest as a marker of intrathecal immunoglobulin synthesis. However, data on inter-laboratory agreement of these measures is lacking. The aim was to assess the concordance of CSF and serum KFLC measurements, and of K-index values, across different laboratories. KFLC and albumin of 15 paired CSF and serum samples were analyzed by eight participating laboratories. Four centers used Binding Site instruments and assays (B), three used Siemens instruments and assays (S), and one center used a Siemens instrument with a Binding Site assay (mixed). Absolute individual agreement was calculated using a two-way mixed effects intraclass correlation coefficient (ICC). Cohen’s kappa coefficient (k) was used to measure agreement on positive (≥5.8) K-index values. There was an excellent agreement in CSF KFLC measurements across all laboratories (ICC (95% confidence interval): 0.93 (0.87–0.97)) and of serum KFLC across B and S laboratories (ICC: 0.91 (0.73–0.97)), while ICC decreased (to 0.81 (0.53–0.93)) when including the mixed laboratory in the analysis. Concordance for a positive K-Index was substantial across all laboratories (k = 0.77) and within S laboratories (k = 0.71), and very good (k = 0.89) within B laboratories, meaning that patients rarely get discordant results on K-index positivity notwithstanding the testing in different laboratories and the use of different platforms/assays.

## 1. Introduction

Multiple sclerosis (MS) is a chronic inflammatory and neurodegenerative disorder of the central nervous system (CNS). An early and accurate diagnosis is particularly relevant for patient management since disease-modifying therapies are most effective at the early stages of the disease [1].

Diagnostic criteria for MS require the demonstration of dissemination in time (DIT) and space (DIS) of demyelinating lesions within the CNS and the exclusion of other possible diagnoses, associated with at least one clinical episode of neurological dysfunction. According to the 2017 revisions of the McDonald diagnostic criteria for MS, the diagnosis of this disease is based on clinical symptoms, imaging by MRI and laboratory testing including CSF examination. The detection of CSF IgG oligoclonal bands (OCB) plays an important role as it permits the substitution of the DIT criterion [2].

Intrathecal immunoglobulin synthesis is a hallmark of MS. Using isoelectrofocusing (IEF), OCB can be detected in up to 95% of cases with clinically definite MS andin 68–83% of patients with a first demyelinating event of the CNS, a clinically isolated syndrome (CIS) [3,4,5]. On the other hand, OCB may be present also in other inflammatory CNS disorders, and therefore, this test is not specific for MS [4,6].

The current laboratory methods for the detection of intrathecal immunoglobulin synthesis are either quantitative, such as the IgG-Index (CSF IgG/Serum IgG divided by CSF albumin/Serum albumin), or qualitative, through the visual detection of OCB on post-IEF, IgG-specific immunoblot. The latter is considered the gold standard [2,7]. Both methods, however, have weaknesses. IgG-Index in MS diagnostics is known to have a low sensitivity [8], while IEF is time-consuming, expensive and, due to its visual interpretation, prone to interrater variability [9,10,11].

B-lymphocytes and plasma cells synthesize a large amount of kappa (KFLC) and lambda (LFLC) free light chains during immunoglobulin assembly [12]. In a healthy state, light chains are produced in slight excess over heavy chains, and the level of unbound light chains, or FLC, in serum and CSF is low. The production of FLC might be abnormally enhanced under pathological conditions such as inflammatory diseases. If an intrathecal synthesis of any immunoglobulin class, whether polyclonal, or oligoclonal, is present, an increase of CSF FLC over serum FLC can be demonstrated [13,14,15], with regard to both KFLC and LFLC. Several studies [11,16,17,18,19,20,21,22,23,24] have demonstrated that CSF KFLC are more sensitive and specific for MS and other inflammatory CNS diseases compared to CSF LFLC.

The kappa index (K-Index), calculated by dividing the CSF KFLC/serum KFLC divided by the CSF albumin/serum albumin, which, in turn, measures the blood-CSF barrier permeability, is gaining increasing interest as a marker of intrathecal activation of the humoral immune response [4,17,25,26,27]. This is of particular relevance in the diagnostic work-up of suspected MS. 

Several studies have assessed intrathecal KFLC synthesis using the K-Index or a hyperbolic reference range (quotient diagrams), demonstrating a predictive value similar to OCB for a MS diagnosis [13,21,22,28,29,30,31], its promise lying also in the automated, low-cost, rapid and objective, quantitative nature of the laboratory method. However, data on the inter-laboratory agreement in CSF and serum KFLC measurements obtained with different instruments is lacking [32]. 

The aim of the present study was to assess the inter-laboratory agreement among laboratories using different platforms—immunonephelometry or immunoturbidimetry—in the determination of CSF/serum KFLC concentrations and of K-Index.

## 2. Materials and Methods

### 2.1. Study Design

Serum and CSF from patients undergoing venous sampling and spinal tap were collected and stored at −80 °C. At the time of the diagnostic procedure, patients consented to biobank storage and future research purposes of the biological samples (Ethics Committee approval nr. 967/2017).

Subsequently, 15 pools of serum and 15 pools of corresponding CSF were made using serum and CSF with similar K-Index values, until a volume of approximately 5 mL of serum and 5 mL of CSF was reached. Samples were selected in order to ensure both high (>10) and very low (1–2) K-index values, as well as K-index values around the cut-off value of 5.8. Samples originated from a total of 37 patients. None of them had been treated with immunomodulatory treatments or had renal function impairment [33]. Diagnoses were Multiple Sclerosis/inflammatory CNS diseases (nr = 20) or, in cases of lower or unmeasurable CSF KFLC values, normal pressure hydrocephalus (nr = 2), polyneuropathy (nr = 5), Guillain-Barré Syndrome (nr = 2), dementia (nr = 2) or meningo/encephalitis (nr = 6). The 15 pairs obtained were then divided into aliquots of about 0.5 mL and sent in dry ice to the 8 Italian centers participating in the study.

Out of the eight participating laboratories, four used turbidimeter and reagents The Binding Site (Birmingham, UK) (B), three used Siemens Healthineers (Erlangen, Germany) nephelometer and reagents (S) and one used the Siemens Healthineers instrument with The Binding Site reagents (mixed). Each laboratory used the same platform and reagent batches for all measurements.

### 2.2. Immunoturbidimetry

Turbidimetric assay was performed by Optilite (Binding Site). The determination of soluble antigen concentration by turbidimetric methods involves the reaction with specific antiserum to form insoluble antigen–antibody complexes. When light passes through the suspension formed, a portion of the light is transmitted and focused onto a photodiode by an optical lens system. The amount of transmitted light is directly proportional to the specific protein’s concentration in the sample. Concentration is automatically calculated by reference to a calibration curve stored within the instrument. As a kinetic reaction, 7 readings are taken at different times and coded by software that automatically obtains the sample concentration and detects excess antigen—that could produce falsely low results—based on the reaction rate. 

The reagent consists of a sheep polyclonal monospecific antibody, and to enhance the reaction, the antibody is coated onto a polystyrene latex particle, allowing amplification of the signal. The Binding Site reagents for Optilite is Freelite Mx Kappa Free Kit for both CSF and serum (range 0.33–127 mg/L). Freelite reacts only with exposed free light chain epitopes, which are hidden when the light chain is bound to the heavy chain. As for albumin, the Low-Level Albumin kit for CSF (linearity range 11–333 mg/L) and Albumin kit for serum (linearity range 2000–66,500 mg/L) were used.

### 2.3. Immunonephelometry

Nephelometric assay was performed with BN II System (Siemens Healthineers). The antigen–antibody reaction produces an insoluble immunocomplex whose scattered light is measured at a fixed angle of 13–24 degrees and with fixed-time kinetics end-point measurement. These aggregates scatter a beam of light passed through the sample. The intensity of the scattered light is proportional to the concentration of the respective protein in the sample. The result is evaluated by comparison with a standard of known concentration. The instrument for FLC assays uses built-in pre-reaction protocols for the detection of antigen excess to avoid false-low test results.

The reagent is based upon the principles of particle-enhanced immunonephelometry. Siemens reagent is N Latex FLC kappa assay (linearity range 0.034–110 mg/L) in both CSF and serum and consists of a suspension of polystyrene particles coated with murine monoclonal antibodies against human KFLC. As for albumin measurement, the N Albumin antiserum anti-human albumin (linearity range 17.0–110,000 mg/L) in CSF and serum was used.

### 2.4. Statistical Analysis

Values were expressed as median and interquartile ranges (IQR). Differences in CSF/serum KFLC and CSF/serum albumin measurements among different laboratories were sought using the Kruskal–Wallis test followed by post hoc tests. Absolute individual agreement between laboratories was calculated using a two-way mixed effects intraclass correlation coefficient (ICC). Lin’s concordance correlation coefficient for CSF and serum KFLC measurements was applied to pairs of laboratories [34]. Cohen’s kappa coefficient was used to measure inter-laboratory agreement on positive (≥5.8) kappa index values. This cut-off was chosen on the basis of a previous study conducted in our laboratory and on literature data [17,23]. Analysis was performed using STATA16 software (StataCorp LLC, College Station TX, USA). *p* < 0.05 was considered statistically significant. 

## 3. Results

**Figure 1:**
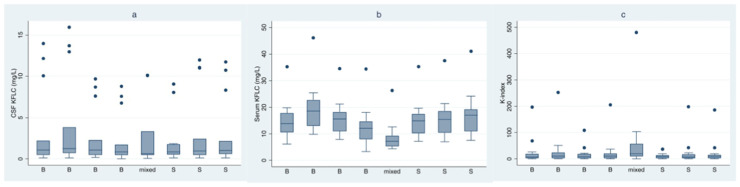
Box-plots showing median (±IQR) CSF (**a**) and serum (**b**) KFLC concentrations (mg/L), and K-index (CSF KFLC/Serum KFLC)/(CSF Albumin/serum Albumin) values (**c**) of the 15 samples across laboratories using Binding Site instruments and assays (B), Siemens instruments and assays (S) or a Siemens instrument coupled with a Binding Site assay (mixed). Upper whiskers show the upper adjacent value (UAV) (largest observation ≤ (third quartile + 1.5 × IQR)); lower whiskers show the lower adjacent value (LAV) (smallest value ≥ (lower quartile − 1.5 × IQR)); black dots show outliers (greater than the UAV or lower than the LAV).

Median CSF/serum KFLC concentrations, K-index values (Figure 1) and CSF/serum albumin measurements (Figure S1) were calculated for the 15 CSF and serum samples determined in each laboratory.

There were no statistically significant differences in overall CSF KFLC and albumin measurements, nor of K-index values, among laboratories. Serum KFLC and albumin levels, however, differed significantly (*p* < 0.001 and *p* = 0.003, respectively) among laboratories, with only a few pairs of laboratories accounting for the difference at post hoc analysis. In particular, serum KFLC levels differed between the mixed laboratory (7.21 mg/L IQR: 5.32–9.34) versus three S laboratories (14.9 mg/L, IQR: 10.2–17.5; 15.37 mg/L, IQR: 10.45–18.58; 17.02 mg/L, IQR: 11–19.2) and two B laboratories (18.56 mg/L, IQR: 13.02–22.72; 15.6 mg/L, IQR: 11.18.2) and serum albumin levels differed between a S laboratory (3590 mg/dL, IQR: 3380–3890) and both a B (4090 mg/dL, IQR: 3800–4470) and the mixed (4145 mg/dL, IQR: 3825–4505) laboratory. Table 1 shows the intraclass correlation coefficient (ICC) for CSF and serum KFLC concentrations and for K-index among B laboratories, among S laboratories, and among B + S and among all laboratories, including the mixed one. Briefly, the ICC is greater for CSF KFLC and albumin as opposed to serum measurements, and ICC decreases for serum KFLC and K-index measurements when the mixed laboratory is included in the analyses.

**Figure 2:**
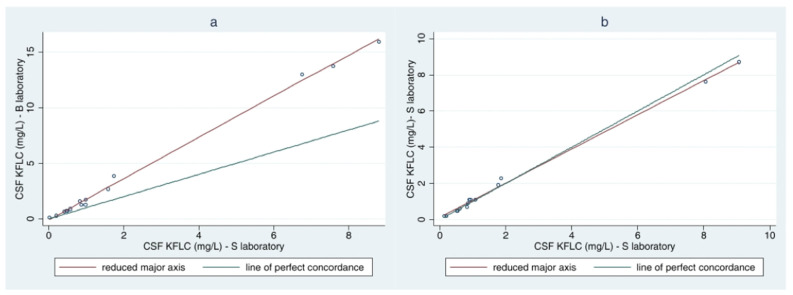
Lin’s concordance correlation coefficient for CSF KFLC measurements in the least concordant (**a**) pair of laboratories (0.769 (95%CI: 0.666–0.873)) and in the best concordant pair (**b**) (0.997 (95%CI: 0.994–0.999)).

Lin’s concordance correlation coefficient ranged from 0.769 (95%CI: 0.666–0.873) between a B and an S laboratory, to 0.997 (95%CI: 0.994–0.999)], between two S laboratories, for CSF KFLC measurements (Figure 2) and from 0.403 (95%CI: 0.213–0.593), between a B and the mixed laboratory, to 0.991 (95%CI: 0.984–0.999), between two S laboratories, for serum KFLC measurements. Cohen’s kappa coefficient for a positive K-index (≥5.8) was 0.89 across B laboratories, 0.71 across S laboratories, and 0.77 across all laboratories. In particular, of the 15 samples analyzed at the eight laboratories, in 11 samples all laboratories gave concordant positive or negative findings, in one case one laboratory gave a discordant negative result (4.6), in one case one laboratory gave a discordant positive result (9.8), and in two cases, more than one laboratory gave discordant negative results, with K-index values (between 4.8 and 5.5), however, quite near the chosen cut-off of 5.8 (Table 2). The KFLC intrathecal fraction and the local KFLC concentrations according to Reiber’s diagram for KFLC [29] are shown in Tables S1 and S2.

## 4. Discussion

The K-index is gaining increasing interest as a possibly more sensitive marker of intrathecal immunoglobulin synthesis compared to OCB in the diagnostic work-up of MS and other CNS inflammatory diseases [25,26,27,28,35,36]. However, several issues remain unresolved: the absence of a recognized international FLC reference standard, scarce data on the CSF matrix stability, several influencing factors on KFLC concentration, e.g., blood contamination, storage conditions, used sampling tubes and, finally, the lack of external quality control [14,32,37,38]. 

The results obtained in this study demonstrate an excellent agreement in CSF KFLC measurements across all laboratories, notwithstanding the use of different platforms and assays. Serum KFLC measurements were slightly less concordant, especially when including the laboratory using a S instrument coupled with a B assay in the analyses. Serum albumin measurements, which in turn influence the K-index calculation, showed less concordance compared to CSF albumin measurements (ICC of 0.79 versus 0.97, respectively), even though the external interlaboratory quality control by INSTAND (Düsseldorf, Germany) [39] shows a good agreement among the serum albumin measurements on the different immunochemistry analyzers [40]. Nevertheless, K-Index values showed an excellent agreement among B and S laboratories and, in particular, among laboratories using the same instrument and assay (B or S); this may be explained by the fact that K-index is a ratio of values obtained from the same instrument and this eliminates the bias that occurs when comparing absolute values.

Currently, the only certificate platforms for CSF FLC assays are two: B and S, based on different reaction principles. B, the oldest platform, on the market since 2001, uses a turbidimeter and a polyclonal antibody-based assay; S, launched on the market ten years later, in 2011, is a nephelometer and uses a monoclonal antibody-based assay. Various authors have highlighted the critical issues related to the detection of FLC [37,38,41,42,43,44]. These methods rely on different calibrators, different analytical methods, different reference ranges and have a different sensitivity. Finally, FLC concentrations in normal CSF are often so low that they cannot be measured despite latex particle enhancement [38].

Despite the highlighted issues, and notwithstanding the low number of samples included and the possibility of greater sampling variability, the results of our study show an excellent agreement in CSF and serum KFLC measurements, in particular among laboratories using either a B analyzer coupled with a B assay or an S instrument coupled to an S assay. Several authors have evaluated serum KFLC data obtained from the two platforms B and S; they concluded that B yields higher values compared to S or, vice versa, S yields lower values compared to B: as no international standard exists an exact value cannot be established [45,46,47,48]. These discrepancies, however, are found mainly in the context of very high serum FLC values, as can be found in patients with multiple myeloma. Patients with lymphoproliferative diseases were not included in our study and all obtained serum KFLC values were within normal limits: this may explain the good agreement between the values obtained from both platforms in the present study. With regard to CSF KFLC measurements, Zeman et al. compared five methods, including nephelometry and turbidimetry, for FLC quantification. Their results are very similar to ours: they found a good correlation between nephelometry and turbidimetry, which was higher for CSF KFLC (rho = 0.979) than for serum KFLC (rho = 0.865) measurements. Furthermore, the observed differences did not impact the prediction of oligoclonal FLC which was considered the reference method [41]. 

The only laboratory using a combined solution, S analyzer and B assay seemed to underestimate serum KFLC measurements which, in turn, increased K-index values, but no conclusions can be drawn at this point, and Süße et al. [49], on the other hand, describe a good agreement between S and B assays used on the same S platform. In particular, the study analyzed 69 samples and showed a good agreement in Passing–Bablok regression analysis (R^2^ = 0.94 for CSF KFLC, R^2^ = 0.88 for serum KFLC).

## 5. Conclusions

The findings of the present study are encouraging, as they demonstrate an excellent agreement in CSF KFLC and a very good agreement in serum KFLC measurements among participating laboratories using different platforms and assays. However, the institutionalization of KFLC external quality control and the increase in the number of participating laboratories is needed in order to ensure harmonized and comparable data for the benefit of clinicians and patients.

## Figures and Tables

**Table 1:** Intraclass Correlation Coefficient (ICC) measured for kappa free light chain (KFLC) and albumin concentrations in cerebrospinal fluid (CSF) and serum and for K-Index = (CSF KFLC/Serum KFLC)/(CSF Albumin/serum Albumin); 95%CI: 95% confidence interval.

ICC	CSF KFLC (95%CI)	Serum KFLC (95%CI)	K-Index (95%CI)	CSF Albumin (95%CI)	Serum Albumin (95%CI)
Binding Site platform and assay (B) (nr = 4)	0.96 (0.9–0.98)	0.93 (0.63–0.98)	0.97 (0.94–0.99)	1.00 (0.99–1)	0.81 (0.41–0.94)
Siemens platform and assay (S) (nr = 3)	0.99 (0.97–100)	0.93 (0.48–0.98)	0.95 (0.89–0.98)	0.99 (0.98–1)	0.93 (0.8–0.98)
B + S (nr = 7)	0.93 (0.85–0.97)	0.91 (0.73–0.97)	0.91 (0.82–0.96)	0.98 (0.96–0.99)	0.83 (0.58–0.94)
All laboratories (nr = 8)	0.93 (0.87–0.97)	0.81 (0.53–0.93)	0.65 (0.43–0.84)	0.97 (0.96–0.99)	0.79 (0.55–0.92)

**Table 2:** K-index measurements across laboratories using Binding Site instruments and assays (B), Siemens instruments and assays (S) or a Siemens instrument coupled with a Binding Site assay (mixed). Discordant results, considering a cut-off for a positive K-index of 5.8, in bold.

Sample	B1	B1	B3	B4	mixed	S1	S2	S3
1	197.0	252.1	108.3	205.3	479.5	170.5	197.9	185.6
2	6.9	6.4	**4.6**	11.8	10.5	5.8	6.0	6.7
3	1.6	2.5	2.0	2.6	4.8	2.3	1.3	1.4
4	7.0	8.6	**5.0**	14.1	18.4	**5.1**	6.3	6.4
5	19.3	24.3	16.0	15.5	57.0	14.4	18.0	14.8
6	3.4	3.1	2.4	3.2	3.6	2.2	2.7	3.4
7	68.5	51.4	42.3	36.5	103.8	36.5	42.8	42.1
8	18.2	24.6	21.1	21.3	42.3	18.8	17.5	15.4
9	19.2	17.6	11.5	10.1	22.7	12.2	15.6	11.1
10	1.8	2.9	3.2	0.4	**9.8**	3.2	2.5	2.3
11	7.7	10.8	9.6	11.5	19.3	10.3	7.8	10.2
12	27.1	28.5	18.6	20.0	86.5	19.3	23.1	20.1
13	1.1	1.2	1.2	1.2	1.2	1.0	1.0	0.9
14	**4.8**	8.2	**5.5**	7.0	14.2	7.5	**5.5**	6.1
15	7.0	12.4	9.5	9.7	23.0	13.5	8.4	8.7

## Data Availability

The data presented in this study are available on request from the corresponding authors.

## Supplementary Materials

The following are available online at https://www.mdpi.com/article/10.3390/biom12050677/s1. Figure S1: Box-plots showing median (±IQR) CSF (mg/dL) (a) and serum (g/L) (b) albumin concentrations (mg/L) of the 15 samples across laboratories using Binding Site instruments and assays (B), Siemens instruments and assays (S) or a Siemens instrument coupled with a Binding Site assay (mixed). Upper whiskers show the largest observation ≤ (third quartile + 1.5 × IQR); lower whiskers show the smallest value ≥ (lower quartile + 1.5 × IQR); black dots show outliers. Table S1: KFLC intrathecal fraction in relation to Qmean according to Reiber’s diagram across laboratories using Binding Site instruments and assays (B), Siemens instruments and assays (S) or a Siemens instrument coupled with a Binding Site assay (mixed). Calculations were done using the free software available at www.albaum.it (accessed on 18 March 2022). Table S2: Local KFLC concentrations according to Reiber’s diagram across laboratories using Binding Site instruments and assays (B), Siemens instruments and assays (S) or a Siemens instrument coupled with a Binding Site assay (mixed). Calculations were done using the free software available at www.albaum.it (accessed on 18 March 2022).
