# Supplementary material for: Inter-Laboratory Concordance of Cerebrospinal Fluid and Serum Kappa Free Light Chain Measurements
Source: Biomolecules. 2022 May 7;12(5):677. doi: 10.3390/biom12050677 (PMC9138559; doi:10.3390/biom12050677)
Supplement: Supplementary file 1 [file biomolecules-12-00677-s001.zip › Supplementar material.pdf]

Manuscript ID: biomolecules-1668946
Type of manuscript: Article
Title: Inter-laboratory concordance of cerebrospinal fluid and serum kappa free light chain measurements
Authors: Patrizia Natali, Roberta Bedin, Gaetano Bernardi, Elena Corsini, Eleonora Cocco, Lucia Schirru, Ilaria Crespi, Marta Lamonaca, Arianna Sala, Cinzia Nicolò, Massimiliano Di Filippo, Alfredo Villa, Viviana Nociti, Teresa De Michele, Paola Cavalla, Paola Caropreso, Francesca Vitetta, Maria Rosaria Cucinelli, Matteo Gastaldi, Tommaso Trenti, Patrizia Sola, Diana Ferraro *

SUPPLEMENTARY

Figure S1: Box-plots showing median (± IQR) CSF (mg/dl) (a) and serum (g/L) (b) albumin concentrations (mg/L) of the 15 samples across laboratories using Binding Site instruments and assays (B), Siemens instruments and assays (S) or a Siemens instrument coupled with a Binding Site assay (mixed). Upper whiskers show the largest observation ≤ (third quartile + 1.5 × IQR); lower whiskers show the smallest value ≥ (lower quartile + 1.5 × IQR); black dots show outliers.

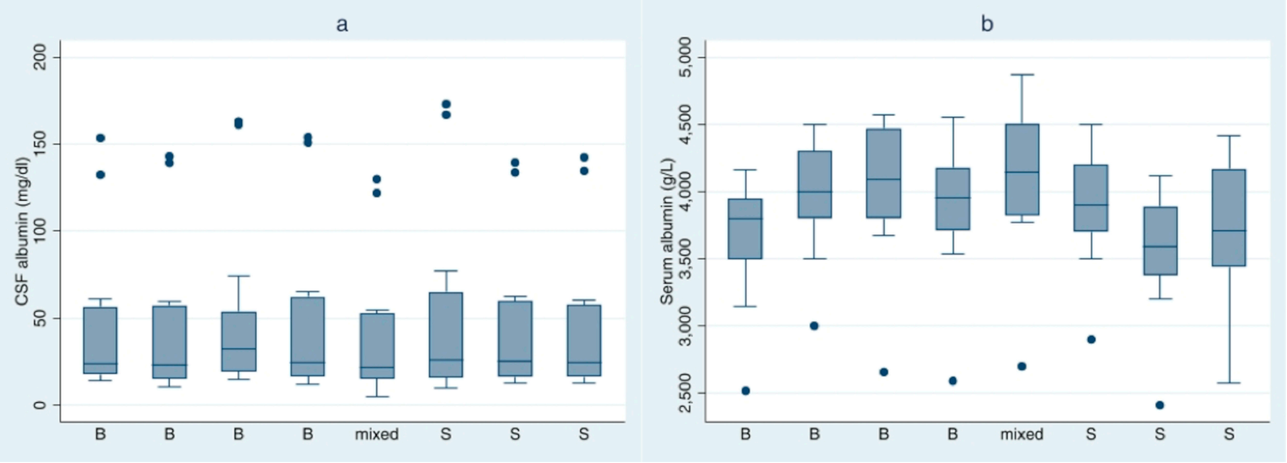

Table S1 KFLC intrathecal fraction in relation to Qmean according to Reiber's diagram across laboratories using Binding Site instruments and assays (B), Siemens instruments and assays (S) or a Siemens instrument coupled with a Binding Site assay (mixed). Calculations were done using the free software available at www.albaum.it.

**Samples**	**B1**	**B2**	**B3**	**B4**	**mixed**	**S1**	**S2**	**S3**
**1**	99.0	99.2	99.0	98.3	99.0	98.8	99.5	98.9
**2**	74.6	72.5	70.7	61.5	85.1	69.9	83.3	74.0
**3**	0.0	20.5	0.0	3.4	24.4	14.8	57.4	0.0
**4**	74.7	79.4	72.0	64.5	87.4	64.8	90.4	72.2
**5**	90.8	92.7	90.2	89.0	88.7	87.8	96.9	88.0
**6**	45.3	40.2	33.3	24.8	42.5	16.0	49.9	45.8
**7**	97.4	96.6	95.9	95.9	95.2	95.2	98.3	95.8
**8**	89.7	92.2	89.5	90.9	91.1	90.1	95.3	88.0
**9**	90.8	90.0	88.6	84.6	82.4	85.5	92.2	84.1
**10**	0.0	5.9	9.6	30.7	0.0	10.3	50.1	1.5
**11**	75.3	82.3	75.7	80.4	83.3	81.8	89.5	81.1
**12**	91.9	92.2	90.9	89.3	89.1	88.6	97.3	89.1
**13**	0.0	0.0	0.0	0.0	0.0	0.0	0.0	0.0
**14**	53.5	71.7	61.0	62.5	67.5	69.2	82.9	63.6
**15**	69.1	81.2	74.6	77.9	77.5	83.2	89.7	74.5

Table S2 Local KFLC concentrations according to Reiber's diagram across laboratories using Binding Site instruments and assays (B), Siemens instruments and assays (S) or a Siemens instrument coupled with a Binding Site assay (mixed). Calculations were done using the free software available at www.albaum.it.

**Samples**	**B1**	**B2**	**B3**	**B4**	**mixed**	**S1**	**S2**	**S3**
**1**	10.0	15.8	11.9	9.5	8.7	9.0	10.1	11.6
**2**	0.4	0.5	0.4	0.3	0.4	0.4	0.4	0.5
**3**	-0.0	0.1	-0.1	0.0	0.0	0.0	0.1	-0.1
**4**	0.9	1.4	0.9	0.7	0.9	0.7	1.2	0.9
**5**	2.0	3.6	2.2	2.0	1.5	1.6	3.2	1.9
**6**	0.5	0.5	0.3	0.2	0.4	0.1	0.2	0.5
**7**	13.6	13.2	10.5	8.3	7.2	8.6	9.9	10.3
**8**	1.5	2.5	1.7	1.7	1.4	1.6	2.0	1.5
**9**	11.0	11.7	9.8	6.4	5.6	6.9	9.3	7.0
**10**	-0.0	0.0	0.0	0.1	-0.1	0.0	0.0	0.0
**11**	0.4	0.6	0.4	0.5	0.4	0.5	0.4	0.5
**12**	1.0	1.5	1.1	1.0	0.7	0.8	1.9	0.9
**13**	-0.7	-0.7	-0.8	-0.7	-0.5	-0.8	-0.3	-1.0
**14**	0.2	0.5	0.3	0.3	0.3	0.4	0.4	0.3
**15**	0.4	0.7	0.4	0.5	0.4	0.7	0.6	0.5
